# Persistent post-stroke depression in mice following unilateral medial prefrontal cortical stroke

**DOI:** 10.1038/tp.2016.124

**Published:** 2016-08-02

**Authors:** F Vahid-Ansari, D C Lagace, P R Albert

**Affiliations:** 1 Ottawa Hospital Research Institute (Neuroscience), University of Ottawa Brain and Mind Research Institute, Ottawa, ON, Canada; 2 Department of Cellular and Molecular Medicine, University of Ottawa, Ottawa, ON, Canada

## Abstract

Post-stroke depression (PSD) is a common outcome following stroke that is associated with poor recovery. To develop a preclinical model of PSD, we targeted a key node of the depression–anxiety circuitry by inducing a unilateral ischemic lesion to the medial prefrontal cortex (mPFC) stroke. Microinjection of male C57/BL6 mice with endothelin-1 (ET-1, 1600 pmol) induced a small (1 mm^3^) stroke consistently localized within the left mPFC. Compared with sham control mice, the stroke mice displayed a robust behavioral phenotype in four validated tests of anxiety including the elevated plus maze, light–dark, open-field and novelty-suppressed feeding tests. In addition, the stroke mice displayed depression-like behaviors in both the forced swim and tail suspension test. In contrast, there was no effect on locomotor activity or sensorimotor function in the horizontal ladder, or cylinder and home cage activity tests, indicating a silent stroke due to the absence of motor abnormalities. When re-tested at 6 weeks post stroke, the stroke mice retained both anxiety and depression phenotypes. Surprisingly, at 6 weeks post stroke the lesion site was infiltrated by neurons, suggesting that the ET-1-induced neuronal loss in the mPFC was reversible over time, but was insufficient to promote behavioral recovery. In summary, unilateral ischemic lesion of the mPFC results in a pronounced and persistent anxiety and depression phenotype with no evident sensorimotor deficits. This precise lesion of the depression circuitry provides a reproducible model to study adaptive cellular changes and preclinical efficacy of novel interventions to alleviate PSD symptoms.

## Introduction

Focal ischemic stroke can lead to both neurological symptoms, including impairments in sensorimotor function and cognition, and behavioral changes in anxiety, depression and emotionality.^1, 2, 3^ Within 3 months of a stroke, 20–50% of patients are diagnosed with post-stroke depression (PSD) following a focal stroke can persist for >1 year after diagnosis.^4, 5^ Patients with PSD often display both anxiety and depressive symptoms,^6, 7^ leading to decreased quality of life, increased mortality and heightened risk of recurrent stroke or suicide.^8, 9, 10^ PSD is also associated with cognitive decline^11, 12, 13, 14^ and interferes with stroke recovery,^15, 16, 17^ leading to increased hospitalization and health care costs.^18, 19, 20^ In addition to PSD associated with focal ischemia, PSD due to silent strokes (vascular depression) is mainly associated with white matter lesions,^21^ with a 10-fold greater prevalence compared with overt strokes.^22, 23^ When diagnosed, patients with PSD or vascular depression are treated with antidepressants, such as serotonin-selective reuptake inhibitors.^6, 24^ However, these drugs take at least 3–4 weeks to elicit a clinical response and they are only 50% effective in treating anxiety and depression, with only 30% of patients achieving remission.^25^ Thus, there is a need for preclinical models to develop improved treatment strategies.

There are several preclinical models of PSD in rodents and non-human primates that have induced large infarcts involving both cortical and subcortical gray and white matter and have reported ‘depressive-like syndromes' characterized by varying degrees of anxiety, despair and anhedonia.^26^ For example, the middle cerebral artery occlusion (MCAO) model in combination with chronic mild stress evokes stress- and stroke-induced behavioral phenotypes that are amenable to antidepressant medications.^27^ However, the MCAO models are often associated with large and variable lesions affecting large regions of cortex and striatum and thus are confounded by pronounced motor impairments that can interfere with assessments of depression and anxiety. Therefore, one of our goals was to develop a reliable model of PSD that was not associated with motor impairments and that persists over time, to test relevant treatments.

Modeling PSD has been challenging due to the size and variability in the location of stroke in patients diagnosed with PSD.^28^ Specific focal lesions associated with PSD are rarely identified since PSD is difficult to diagnose without overt physical impairment. Although PSD is generally more frequent in subjects suffering from left hemispheric lesions,^12, 29, 30^ recent reports have found that right hemisphere lesions are also associated with PSD during acute post stroke period (⩽3 months).^31^ Clinical studies have additionally demonstrated that PSD is associated with lesions involving cortico-limbic circuitry.^32, 33, 34, 35^ In particular, recent studies support a positive relation between PSD and damage to some basal ganglion or frontal lobe structures including the medial prefrontal cortex (mPFC).^36^ Furthermore, alterations in the activity of the mPFC have been strongly implicated in depression-like behavior,^37, 38, 39^ as well as projections between mPFC and the basal ganglia and the nucleus accumbens.^40, 41^ Given that large infarcts that may impair mPFC function can induce depression and anxiety, we tested whether a small unilateral focal stroke targeted to left mPFC would be sufficient to disrupt affective circuitry and yield a potentially useful model of PSD.

To produce a focal stroke in the mPFC, we used the endothelin-1 (ET-1) model. ET-1 is a vasoconstrictor peptide that when injected results in a discrete ischemic lesion by causing blood vessels constrict acutely to reduce blood flow in the target area.^42^ The ET-1 model was initially characterized in rats as a simple and reproducible method of focal ischemia.^42, 43^ More recently, others have shown in mice that ET-1 can also be used to make a specific lesion of the mouse motor cortex that is associated with consistent locomotor impairments.^44, 45^ In this study, ET-1 microinjection in the left mPFC in male C57/BL6 mice was used to generate a unilateral and reproducible focal ischemic lesion, resulting in a robust anxiety and depression phenotype that persisted for at least 6 weeks without sensorimotor impairment. These findings suggest that the ET-1 lesion to the mPFC may be useful to examine changes in regional brain activity that lead to the PSD phenotype, as well as to develop, test and optimize treatments for PSD.

## Materials and methods

### Animals

Seventy one male C57/BL6, 10–11 weeks old (Charles River Laboratories, Montreal, QC, Canada) weighing 25–28 g at the time of surgery were used in this study. Mice were pair-housed in standard Plexiglass cages on a 12/12 h light/dark cycle with ad libitum access to food and water. Animals were allowed to acclimate to the housing facility for 2 weeks prior to surgery. Forty mice were tested in 2 cohorts of 20 mice for behavioral assays based on published literature for these tests.^46, 47^ For each cohort, the mice were randomly divided into either the sham control (*n*=10) or stroke (*n*=10) group and no blinding was done. For histology, 11 additional mice were used including *n*=3 at 48 h and *n*=4 at 1 week, 2 weeks, *n*=4 per group; for 6 weeks post surgery 4 mice from the behavioral cohort were used. A separate cohort of mice was used to test depression-like behavior at 6 weeks post stroke (sham=10; stroke=10). The University of Ottawa Animal Care Committee approved all experimental procedures in accordance with guidelines established by the Canadian Council of Animal Care.

### ET-1 injection

**Figure 1:**
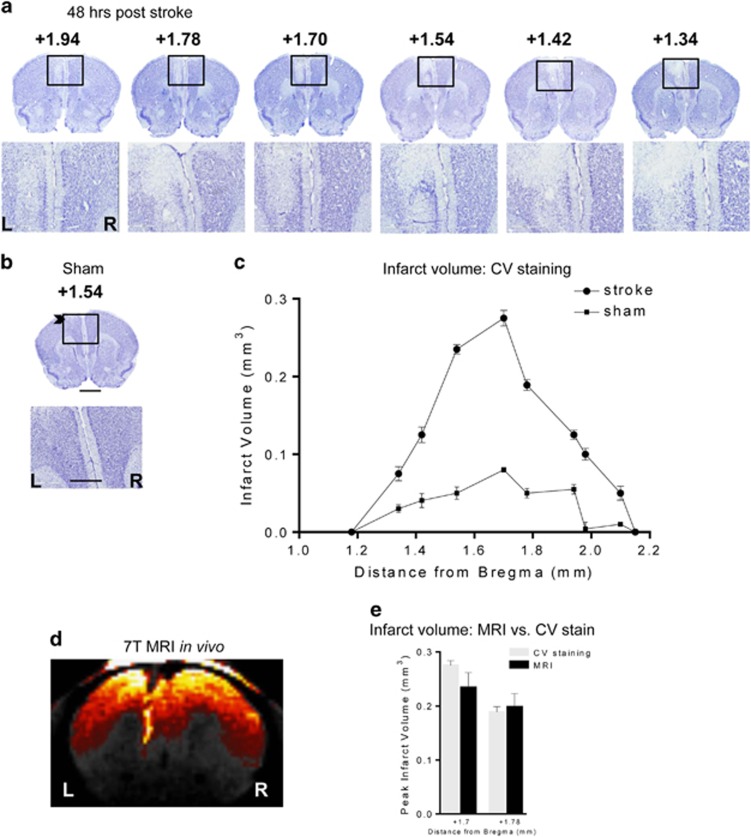
Lesion location following ET-1 injection into left mPFC. (**a**) Representative microphotographs of CV-stained brain sections from a representative ET-1-injected mouse showing cell loss at 48 h post surgery shown at low (× 3.5) and high (× 20) magnification of the lesion site (box) with bregma locations indicated above. Scale bar, 400 and 50 μm. (**b**) A representative microphotograph of a CV-stained section of a sham control showing a trace of the needle track (arrowhead) detectable on left (L) side compared with the right (R) at 3.5 × and 20 × magnification. Scale bar, 400 and 50 μm. (**c**) Quantification of ET-1-induced lesion volumes at 48 h post surgery through the longitudinal axis of the lesion according to bregma locations (sham, *n*=3; stroke, *n*=3). (**d**) ET-1 lesion site visualized *in vivo* by MRI. Shown is a representative 7 T MRI image done in an anaesthetized living mouse at 4 days post stroke showing a 300-μm MRI section in which the lesion site is visualized and limited to the left mPFC (left, L; right, R); *n*=4–5 per experimental group. (**e**) Comparison of lesion size in MRI versus CV staining. Images obtained from *in vivo* MRI scanning or post-mortem CV staining of the same mice were quantified at two different distances from Bregma. Infarct volumes showed low variability and did not differ between the two measures. *n*=4. CV, cresyl violet; ET-1, endothelin-1; mPFC, medial prefrontal cortex; MRI, magnetic resonance imaging.

Mice were anesthetized with isoflurane (5% induction, 1.5–2% maintenance with oxygen 1 l min^−1^) prior to stereotaxic surgery. During surgery, body temperature was maintained between 36.5 and 37.5 °C using a feedback homeothermic heating blanket. ET-1 (Calbiochem, San Diego, CA, USA) was suspended in sterile water for complete solubility (2 μg μl^−1^=800 pmol μl^−1^) by cold sonication at 4 °C for 15–20 min and 1.0 μl was injected using an infusion pump set at a rate of 0.20 μl min^−1^. ET-1 was injected at two sites within the left mPFC at the following coordinates (in mm) relative to Bregma^48^ (Figure 1a): Injection 1, anterior–posterior (AP), 2.0; medial–lateral (ML), +0.5; dorsoventral (DV), −2.4; Injection 2, AP 1.5; ML +0.5; DV −2.6. At the end of the infusion, the needle was left in place for 3 min before gradual withdrawal and closure of the incision that was treated with 0.1 ml of 2% transdermal bupivacaine (Chiron, Guelph, ON, Canada) as a topical anesthetic. Post surgery, the mice were placed in a 37 °C incubator to maintain body temperature until they regained mobility. At ~3 h post surgery, the mice were given a single injection of buprenorphine (0.03 mg kg^−1^ s.c., Reckitt Benckiser Pharmaceuticals, Richmond, VA, USA) for pain management and then returned to their housing room. Sham control mice underwent the same procedures except that the ET-1 was replaced with vehicle (sterile water).

### Cresyl violet staining

At 2, 7, 14 or 42 days post surgery, mice were deeply anesthetized by administration of Euthanyl (149.5 mg kg^−1^ i.p., Bimeda-MTC Health, Cambridge, ON, Canada) and transcardially perfused with phosphate-buffered saline (PBS) 0.1 m, followed by 4% paraformaldehyde (PFA) in PBS, pH 7.4. Brains were removed and post fixed in 4% PFA overnight at 4 °C, then transferred to sucrose 30% (w/v) in 0.1 m phosphate buffer solution until saturated. Coronal sections (25 μm thickness) were cut through the mPFC and collected in 10 serial sections using a microtome. For each animal, every fourth section throughout the mPFC lesion was stained with cresyl violet (CV).^49^

### Infarct volume assessment

Using ImageJ (NIH, Bethesda, MD, USA, http://imagej.nih.gov/ij/), the area of contralateral and intact ipsilateral tissue remaining was measured for every fourth serial coronal section (stained with CV) throughout the lesion in mPFC. Infarct volume in mm^3^ was calculated as follows: Σ (area of contralateral lesion tissue−area of intact ipsilateral tissue) × thickness of the interval between sections.^50^

### Magnetic resonance imaging

Magnetic resonance imaging (MRI) was done to visualize and verify early lesion development *in vivo* using a 7T GE/Agilent MRI machine (Milwaukie, WI, USA) at the University of Ottawa Preclinical Imaging Core Facility (sham, *n*=10, stroke, *n*=10). At 4 days post stroke, a subset of animals (*n*=4–5 per group) was anesthetized with isoflurane in O_2_: induction at 3%, maintenance at 1.5% isoflurane. Serial MRI images were taken from the PFC at 300 μm thickness. A fast spin echo pulse sequence was used with repetition time=4500 ms, effective echo time=13 ms, field-of-view=3 cm, matrix size=256 × 256, slice thickness=300 μm, number of averages=2, axial (transverse) image orientation and scan time=6 min. Following MRI, the animals were placed in their home cage and observed until recovery from anesthesia and then returned to the housing room. Results of MRI imaging were also verified initially using TTC staining performed immediately after scanning on same animals. Briefly, three to four serial coronal sections (1-mm thickness) were prepared and stained in saline containing 2% TTC (Sigma) at room temperature (RT)/dark for 30 min (data not shown) and the infarct size was classified as small as defined previously (<20% of the hemisphere).^51^

### Immunofluorescence

Coronal brain slices (25-μm thickness) of mPFC including cingulate cortex (CG), infra- and pre-limbic mPFC (coordinates; Bregma +1.70 to +1.54 mm)^48^ were obtained from brain tissue from PSD mice at 48 h (*n*=3) and 6 weeks (*n*=3) post stroke. The sections were washed 3 × in PBS, blocked 1 h in PBS with 1% BSA, 10% NDS, 0.1% Triton X-100 followed by 3-h incubation at RT with rabbit anti-IBa1 1:1000 (Wako, Richmond, VA, USA, 019–19741), and goat anti-GFAP 1:500 (Santa Cruz, Dallas, TX, USA, sc-6170) and mouse anti-NeuN 1:1000 (Millipore, Mississauga, ON, Canada, MAB377). Sections were washed three times in PBS followed by 1-h incubation at RT with Alexa Fluor 488 donkey anti-rabbit IgG, 1:1000 (Life Technology, Beverly, MA, USA, A-21206); donkey anti-goat Cy5, 1:250 (Life Technology, A-21447) and Alexa Fluor 594 donkey anti-mouse IgG, 1:200 (Thermofisher, Mississauga, ON, Canada, A-21203) in blocking solution. Images of left mPFC were acquired with the Axiovision imaging software (Carl Zeiss Canada, North York, ON, Canada) on a Zeiss Axio Observer D1 microscope (Carl Zeiss Canada).

### Behavioral assays

Several validated behavioral tests measuring anxiety, depression and sensorimotor deficits were performed at the UOttawa Behavioral Core Facility in littermates following a unilateral PFC stroke. Mice were housed under normal light conditions and tests were performed beginning at 1000 hours, 2 h after lights were turned on and at least 1 h after the mice were habituated to the testing room. Testing was performed under white light illumination with the exception of the forced swim test (FST) and cylinder test (Cyl), which were performed under red light. All animals were of the same age at the start of testing, and behavioral testing began at 1 week post surgery and lasted up to 6 weeks post surgery, two cohorts *n*=10 per group. Tests used included the elevated plus maze (EPM), open field (OF), FST, tail suspension (TS), novelty-suppressed feeding (NSF), beam break (BBK), horizontal ladder, Cyl and light–dark test (LD) that were performed according to the timeline presented (Figure 4a), although not all tests were done in each cohort as indicated by *n*-values in figure legends. One additional cohort (*n*=10 per group) was used to test for FST at 6 weeks post stroke. Throughout testing and behavioral analyses, experimenters were blind to the treatment groups.

### Elevated plus maze

EPM is a test that assesses anxiety-like behaviors.^52^ The mice were placed in the center of an elevated two-arm plus maze, measuring ~20-cm high, ~6-cm wide and ~75-cm long (Noldus, Wageningen, The Netherlands). The arms of the maze are crossed with one arm having an open platform, the other arm having a closed platform with walls that are ~20-cm tall. Mice were placed in the maze with the head toward the closed arm of maze and allowed to explore the maze for 10 min. The mouse movements were videotaped and the time spent in closed and open arms was analyzed (Ethovision 8, Noldus Information Technologies, Leesburg, VA, USA).

### Open field test

The OFT measures exploration in response to a novel large open arena as a measure of anxiety.^46, 47^ The mice were placed in a corner of the arena (45-cm long in each side and 45-cm high) and allowed to explore the new environment for a total of 10 min at light levels of 300 lux. Mouse movements were videotaped and the time spent in the center (24 × 15 cm) and corners (squares with 10-cm sides) of the OF arena was analyzed (Ethovision 8).

### Light–dark test

The light–dark paradigm is a measure of anxiety based on aversion to bright light and exploratory behavior in a novel environment in the presence of mild stressors.^53^ Mice were placed into the light–dark chamber (Med Associates, St Albans, VT, USA), which is a box divided into two equal areas measuring 27 × 13.5 cm, with the dark area being completely black and covered, whereas the open area is transparent. The chamber is housed in a sound proof box that is additionally equipped with a fan and two bright light bulbs that produce 390 lux white illumination. The mouse is placed into the corner of the open area and allowed to move freely between the two areas for 10 min through an opening between the two chambers. The distance traveled in each zone, the total number of transitions, the time spent in each zone and the latency to enter the light compartment were recorded by using photo beam-based video-tracking software (Med Associates).

### Novelty suppressed feeding test

The NSF test was used to assess anxiety-related behaviors.^54^ Briefly, animals were food deprived for 16 h. After 3 min of habituation they were placed in a new cage. Animals were individually placed in an arena (45-cm long in each side and 45-cm high; 100 lux) with a food pellet placed in the center. The latency of the mice to begin eating food was recorded manually and immediately after mice chewed the food or after 10 min had expired for the trial, the mice were removed from the arena and placed in their home cage and the amount of food consumed in 5 min was measured.

### TS test

The TS test measures stress coping response.^55^ For this test, the mouse TS boxes from Med Associates were utilized and the tail of the mouse was secured with tape to a horizontal bar and the animals were suspended for 6 min. The automated detection device (ENV-505TS Load-Cell Amplifier, Med Associates, Fairfax, VT, USA) was used to determine mobility and immobility time through Med Associates software.

### Forced swim test

The FST is used for assessing antidepressant-like activity.^55^ The animals are subjected to an inescapable swim stress for a short period of time. Briefly, each mouse was placed into clear plastic cylinder 22 cm in diameter and 37-cm deep filled with 4 l of 24 °C water. The mouse was videotaped from the side of the cylinder for 6 min under red lighting and the duration of mobility and immobility time was quantified using Ethovision XT automated video-tracking software from Med Associates.

### Home cage locomotor activity

Mice were placed into a novel home cage for 30 min and activity was measured by number of breaks of an invisible infrared light beams located on a frame surrounding the animal cage using the home cage locomotor activity system^56^ (Omnitech Electronics, Columbus, OH, USA).

### Horizontal ladder test

Horizontal ladder is a locomotor test used to evaluate forelimb and hind limb stepping, placing and coordination.^57^ The animals were trained (1 trial) and tested (3 trials) to walk on a horizontal ladder (composed of two clear Plexiglas walls (69.5 × 15 cm) containing 121 metal rungs (0.15 cm in diameter, 2 cm from bottom of wall), spaced regularly (1 cm apart) or irregularly (0.5–2.5 cm apart)) while being video recorded. The number of successful and slips or missed steps were quantified.

### Cylinder test

The cylinder test is used to assess sensorimotor function of the forelimb.^44^ Mice were placed into a glass cylinder (10 cm diameter, 15-cm high) under red light condition and video recorded. Mice were required to perform a minimum of 20 rears at which time the test ended. The amount of time each limb was used to support the mouse throughout rearing was manually quantified.

### Statistical analyses

All analyses were done using the Statistical Package for the Social Sciences (GraphPad Prism version 6.00 for Windows, GraphPad Software, La Jolla, CA, USA; www.graphpad.com). Data are expressed as mean±s.e.m. Using the ROUT method in GraphPad Prism to identify outliers, one subject in TS and two subjects in EPM and FST, 6 weeks post stroke, were excluded from analyses. The behavioral data for the two cohorts of mice tested were not significantly different and therefore all data shown is the combined data from the two cohorts. Data comparing the sham and stroke mice on one outcome measure were analyzed using an unpaired *t*-test. One-way analysis of variance followed by Dunnett's multiple comparisons test was performed for comparing infarct volume at different time points.

## Results

### ET-1-induced infarct within the mPFC

ET-1 was microinjected sequentially in two locations within the left mPFC and at 48 h post ET-1 injection a focal lesion confined to the left mPFC was observed (Figure 1a). The lesion was significantly different from sham control mice that had only minimal needle track injury (Figure 1b). In the stroke mice, the lesion included all sub-regions of the mPFC including CG, pre-limbic and infra-limbic areas (Figure 1a). Detailed analysis of the location of the lesion through the longitudinal axis of the brain also showed very little variability between mice, and a peak in infarct volume between Bregma +1.54 and +1.78 (Figure 1c). In addition, MRI analysis was done on a subgroup of each cohort used for behavioral studies to visualize and confirm successful and accurate lesion of the left mPFC *in vivo* (Figure 1d). The MRI data showed consistent lesions, with similar average volume to that obtained by CV staining (Figure 1e). Thus, the ET-1-induced lesions were small, precise and reproducible, and no animals died or were excluded.

**Figure 2:**
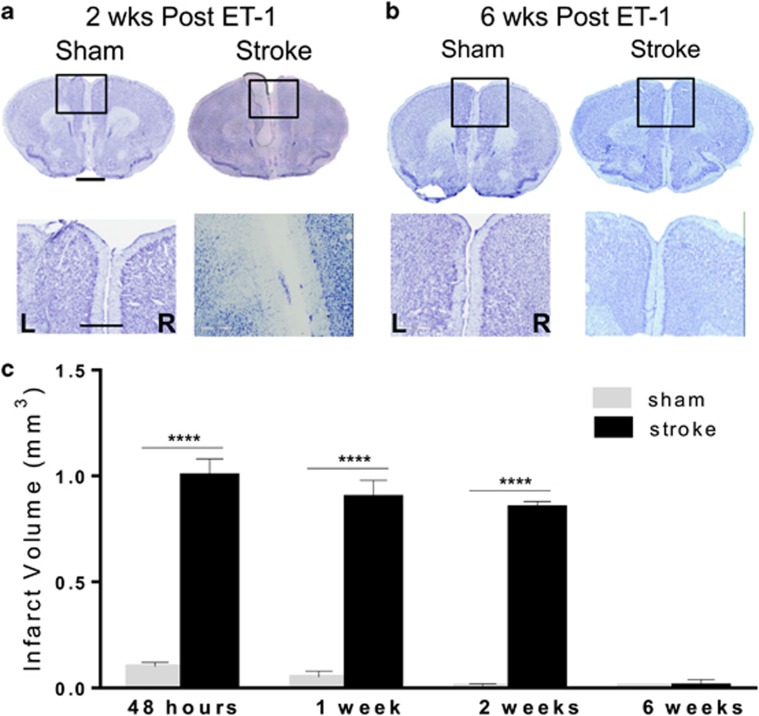
Quantification of infarct volume over time. (**a**–**c**) Quantification of ET-1-induced lesion size by CV staining over time. Representative microphotographs of CV-stained brain sections of sham and ET-1 lesions in left (L) mPFC versus right (R) mPFC at 2 weeks (**a**) or 6 weeks (**b**) post stroke. High magnification (20x) images of the lesion site (box) are shown. At 2 weeks post stroke, an infarct with cell loss restricted to the left mPFC is visible, but is no longer observed at 6 weeks post stroke in any sub-regions of the left mPFC. Scale bar, 400 μm; 50 μm. (**c**) Infarct volume as quantified from CV-stained sections from sham and ET-1-injected mice at 48 h (*n*=3), 1 week (*n*=4), 2 weeks (*n*=4), and 6 weeks (*n*=4) post surgery (mean±s.e.m.) ****P<0.001 sham versus stroke; *P<*0.001 sham group at 6 weeks versus the other sham time points, *P<*0.001, stroke at 6 weeks versus all other stroke time points. CV, cresyl violet; ET-1, endothelin-1; mPFC, medial prefrontal cortex.

**Figure 3:**
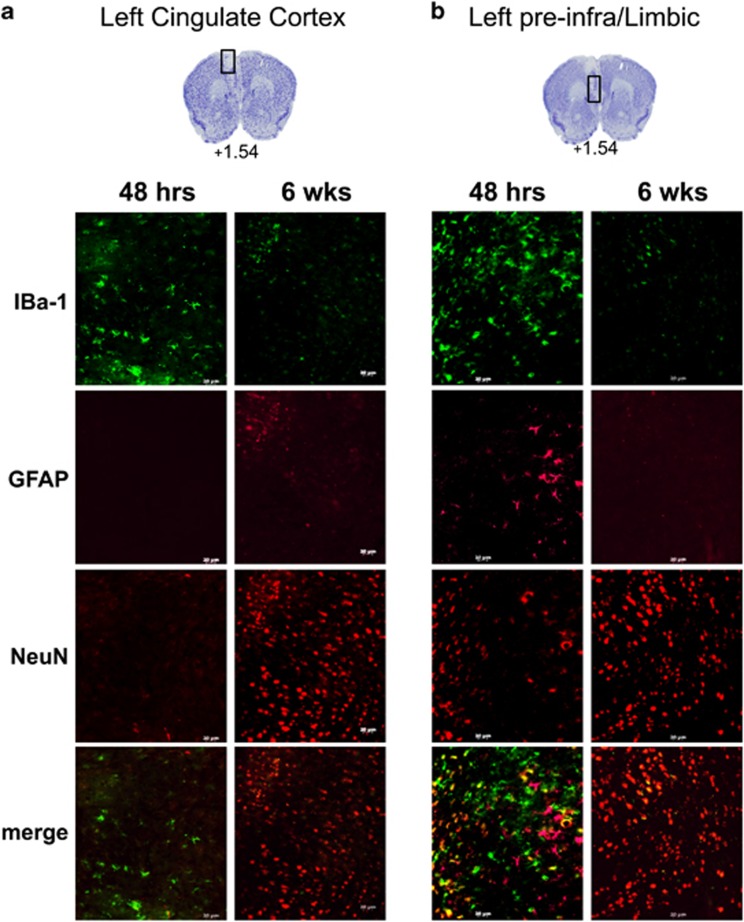
Cellular changes in the ischemic lesion site over time. Brain sections of the left mPFC (Bregma +1.54) were examined by immunofluorescence at the (**a**) cingulate gyrus (CG) and (**b**) pre- and infra-limbic cortex from mice 48 h (left) or 6 weeks (right) following ET-1 microinjection in left mPFC. Representative images of sections stained with anti-IBa1 (microglia), GFAP (astrocyte) and NeuN (neurons). At 48 h post stroke, the lesion site had IBa1-expressing cells in the absence of NeuN-expressing cells (left panels). In contrast at 6 weeks post stroke, the lesion site had NeuN-expressing cells in absence of IBa1 cells (right panels). Scale bar, 20 μm (48 h, *n*=3; 6 weeks, *n*=4). ET-1, endothelin-1; mPFC, medial prefrontal cortex.

The size of the ischemic lesion at later times during which behavior was studied was determined. Examination of the stroke lesion site at 2 weeks post ET-1 injection (Figure 2a) revealed no significant change in lesion volume compared with either 48 h or 1 week post ET-1 injection (Figure 2c). In contrast, the lesion cavity was no longer evident at 6 weeks post stroke (Figures 2b and c). To determine the phenotype of the cells located within the lesion site, brain sections including both left CG (Figure 3a) and limbic cortex (Figure 3b) were analyzed by immunohistochemistry using antibodies for markers expressed in either microglia (IBa-1), astrocytes (GFAP) or neurons (NeuN). At 48 h post stroke, the lesion site had mainly IBa-1- and GFAP-positive cells, with few NeuN-positive cells at the lesion periphery, as shown in Figure 3 (left panels) and quantified in Table 1. By contrast, at 6 weeks post stroke, few IBa-1- or GFAP-positive cells were detected at the periphery of the lesion, while the lesion site was largely populated with NeuN-positive cells (Figure 3, Table 1). These results suggest that the ischemic lesion initially triggers a glial response with activation and invasion of microglia and astrocytes, followed by infiltration of neuronal cells expressing NeuN within the lesion by 6 weeks post stroke.

### Anxiety and depression phenotype following infarct of the left mPFC

**Figure 4:**
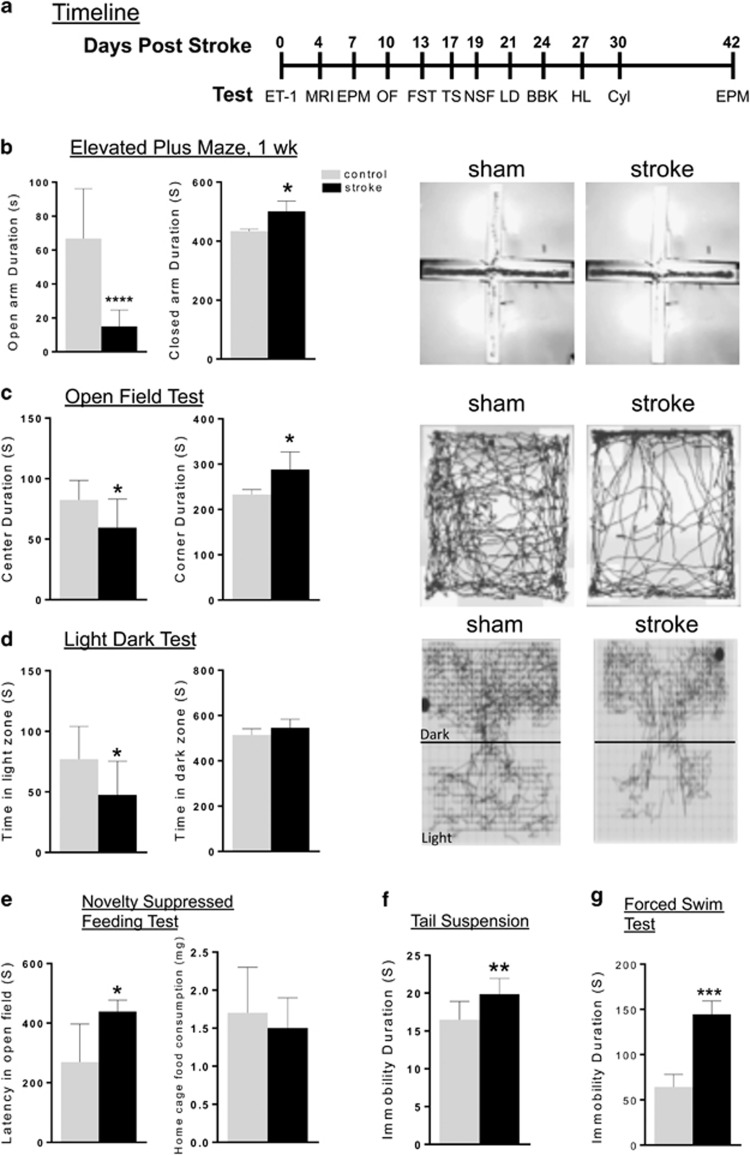
Anxiety and depression phenotype in stroke compared with sham mice. (**a**) Timeline for behavioral studies post stroke. After ET-1-induced lesion of the left mPFC (day 0), a battery of tests was conducted from 1–6 weeks post lesion, including: BBK, beam break; Cyl, cylinder test; EPM, elevated plus maze; FST, forced swim test; HL, horizontal ladder test; LD, light dark; NSF, novelty-suppressed feeding test; OF, open field; TS, tail suspension. One or both cohorts were run on selected tests as indicated. (**b**) EPM: stroke mice spent significantly less time in the open arm and increased time in the closed arm duration compared with sham mice (single mouse tracking shown at right). Sham *n*=20; stroke *n*=20. (**c**) OF: the stroke mice spent significantly less time in center and more time in corner of the open field compared with sham mice (single mouse tracking shown at right). Sham *n*=10; stroke *n*=10. (**d**) LD: the stroke mice spent significantly less time in light zone of the light dark box compared with sham mice (single mouse tracking shown at right). Sham *n*=10; stroke *n*=10. (**e**) NSF: the stroke mice had a longer latency to feed compared with sham mice. The total food consumption in 5 min in the home cage was not different between stroke (*n*=10) and sham mice (*n*=10). (**f**) TS: the stroke mice had significant more immobility compared with sham mice on tail suspension test. (**g**) FST: the stroke mice showed significantly more immobility compared with sham mice on forced swim test. Data represent mean±s.e.m.; sham, *n*=20, stroke, *n*=20. *P<0.05; **P<0.01; ***P<0.001. ET-1, endothelin-1; mPFC, medial prefrontal cortex.

To determine whether unilateral stroke in the left mPFC was sufficient to elicit anxiety- or depression-like behavior or altered motor function, sham and stroke mice were compared using multiple behavioral tests.^58^ Beginning at 1 week after ET-1 injection, the mice were tested for anxiety- and depression-like behavior sequentially, with the least stressful anxiety tests first, then for locomotor function, at times shown in the timeline (Figure 4a).

In each of the behavioral tests, the stroke mice displayed a clear phenotype compared with sham controls. In the EPM test, the stroke mice had a significant 80% reduction in open arm duration (Figure 4b). In the OF test, the stroke mice spent significantly less time in center of the arena and more time in corners of the arena compared with control mice (Figure 4c). In the light–dark test, the stroke mice also had a significant reduction time spent in light zone compared with control mice (Figure 4d). In the NSF test, the stroke mice displayed significantly greater latency to feed in novel cage while similarly consumed food in home cage (Figure 4e). On the TS test, the stroke mice had significantly greater immobility time compared with controls (Figure 4f). Stroke mice also showed significantly greater immobility in the FST compared with control mice (Figure 4g). Overall, these behavioral analyses show a very strong anxiety- and depression-like phenotype in the ET-1-lesioned mice in all tests compared with sham control mice.

### Intact locomotor activity and sensorimotor performance following lesion to the mPFC

**Figure 5:**
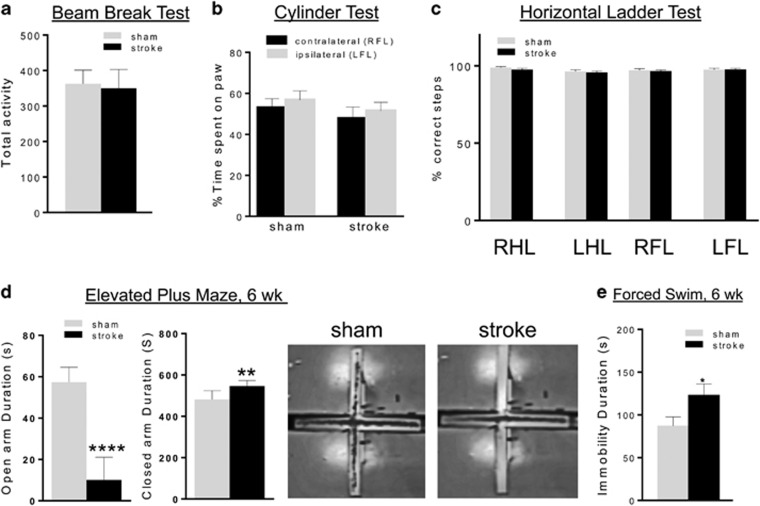
Persistent behavioral phenotype without locomotor impairment in stroke mice. (**a**–**c**) Locomotor/sensorimotor tests. (**a**) Activity test: the total locomotor activity when placed in a novel cage was monitored for 30 min and was not different between sham and stroke mice. (**b**) Cylinder test: no differences in time spent on contralateral and/or ipsilateral paw were detected using the cylinder test between stroke and sham mice. (**c**) Horizontal ladder test: no differences in correct foot placement or foot faults were observed between stroke and sham mice (LFL, left forelimb; LHL, left hind limb; RFL, right forelimb; RHL, right hind limb.). Data represent mean±s.e.m.; sham, *n*=10; stroke, *n*=10. (**d**, **e**) Anxiety and depression tests at 6 weeks post stroke. (**d**) EPM, week 6: after 6 weeks, EPM was repeated and stroke mice displayed reduced open arm and increased closed arm duration compared with sham mice, indicating a persistent anxiety phenotype that does not spontaneously recover (single mouse tracking shown at right). Sham, *n*=20; stroke, *n*=20. (**e**) FST, week 6: in a separate cohort at 6 week post stroke, the stroke mice displayed greater immobility duration compared with sham mice (sham, *n*=10; stroke, *n*=10). *P<0.05; **P<0.01; ****P<0.001. EPM, elevated plus maze; FST, forced swim test.

Because the ET-1-induced lesion was restricted to the left mPFC and did not include the sensorimotor cortex, we hypothesized that locomotor activity and sensorimotor function would not be affected. In agreement with this hypothesis, locomotor activity monitored by total distance moved in both in the EPM (total activity, sham versus stroke; 476±35.8 cm versus 482±20.8 cm) and OF tests (total activity, sham versus stroke; 3789±127.2 cm versus 3579±168.5 cm) (Figures 4b and c) did not differ between stroke and control mice. Furthermore, there was no difference in number of BBKs between the stroke and sham mice up to 30 min after placement into a novel cage during the home cage activity test (Figure 5a). Together, these findings indicate the stroke mice show normal locomotor activity.

Sensorimotor function was assessed using the cylinder and horizontal ladder tests that are highly sensitive to ischemic lesions of sensorimotor cortex.^44, 50, 59^ In the cylinder test, stroke and sham mice spent an equal amount of time with the contralateral (right) and/or ipsilateral (left) forelimb paw on the wall (48 versus 50%) (Figure 5b). Similarly, in the horizontal ladder test the stroke and sham mice made almost no foot faults (about 2%) on the fore- or hind-limbs of ipsi-/contralateral sides (Figure 5c). Together, these tests suggest that the stroke in the mPFC did not induce impairment in sensorimotor function, as seen in silent strokes.

### Persistent behavioral phenotype in stroke mice

Since histological assessment suggested that cellular remodeling of the lesion site had occurred at 6 weeks post stroke, we addressed whether the behavioral phenotype was still present at 6 weeks post stroke. When re-tested in the EPM, the stroke mice showed a significant 80% reduction in open arm time compared with sham mice (Figure 5d), similar to the phenotype observed in these mice at 1 week post stroke (Figure 4b). In a separate cohort of mice tested in the FST at 6 weeks post stroke, the stroke mice displayed about 50% increase in immobility compared with sham (Figure 5e). These results indicate that at 6 weeks post stroke, the stroke mice display both anxiety- and depression-like behavior. The retention of the anxiety and depression phenotype in the stroke mice indicates that the cellular remodeling of the infarct in the left mPFC at 6 weeks post stroke is not associated with functional recovery.

## Discussion

Emotional disturbances associated with PSD, such as lack of motivation, depressed mood, agitation and anxiety, are common after stroke.^3^ Current antidepressant treatments result in remission in only 30% of depressed patients, hence improved preclinical ischemic models are needed to develop and optimize therapies for PSD.^26^ Herein, we have developed a mouse model of PSD using unilateral microinjection of ET-1 into the left mPFC. This results in a very small stroke that is associated with a persistent depression- and anxiety-like phenotype in the absence of motor deficits.

The manifestation of PSD in humans has been associated with lesions located in the mPFC–midbrain–limbic circuitry that is implicated in depression and anxiety.^36, 60, 61, 62^ The PSD mouse model developed here is relevant more generally to PSD following larger overt strokes that affect the PFC–subcortical circuitry. Although strokes restricted to the mPFC are rare in humans, we targeted this region to determine the outcome of an ischemic lesion in the mPFC, given that this region has been implicated in PSD.^61, 62, 63, 64, 65^ We also decided to use mice to generate a model that could exploit the large variety of conditional and inducible gene knockout mouse strains for future studies to further investigate the role of cellular remodeling and circuitry changes in PSD. Our results suggest that unilateral lesion of the PFC is sufficient to induce a robust anxiety and depression phenotype, in the absence of infarct in other cortical and subcortical gray and white matter.

Our model of unilateral mPFC lesion may also have implications in understanding why the prevalence of vascular depression increases with the number of white matter lesions, since these lesions can progressively disrupt connections between the mPFC and subcortical nuclei.^32, 33, 34, 35^ By disrupting the axons, white matter strokes also may induce retrograde damage to projection neurons in the mPFC, including neurons and their axons that are targeted in our unilateral lesion model. However, animal models of white matter lesions leading to anxiety and depressive symptoms are yet to be reported. Although the etiology may differ, our model may be useful for understanding the progression and treatment of silent white matter strokes.

### Unilateral ischemia in the mPFC as a model of PSD

The PSD mouse model we describe was generated by microinjection of ET-1 in the left mPFC. In addition, because a symmetric bilateral stroke would be extremely rare clinically, a unilateral rather than bilateral stroke has greater clinical relevance. The left side was targeted since left MCAO in rodents, and left hemisphere stroke in humans is more strongly associated with PSD, and is followed by other poorer stroke outcomes associated with PSD.^26^ Since this ET-1 ischemia model specifically targets the left mPFC, it has several major advantages compared with the often utilized MCAO stroke model.^26^ With the MCAO model, the stroke lesion is often large, and produces inconsistent infarct size unless occlusion durations are very long, due to inherent inter-animal variations in the collateral blood supply.^26, 66^ In our model, we have obtained very low variability in lesion size and location between mice (Figure 2) and there was no mortality. Unlike some MCAO models, there was no need to combine mPFC lesion with chronic mild stress to observe depression-like behavior.^27^ In addition, the small ET-1-induced lesion was limited to the left mPFC and did not result in detectable locomotor or sensorimotor impairment. In contrast, the MCAO model is generally associated with sensorimotor impairments that can confound standard behavioral measures used to detect emotional function that rely on sensorimotor function, including assays used in this study.^66^ Electrolytic or excitotoxic lesions have previously been used to selectively ablate mPFC^67, 68^ but can have distinct consequences.^69^ However, these lesions do not mimic the key characteristics of an ischemic lesion of a reversible loss of blood supply that may lead to long-term behavioral impairments that are potentially reversible. Thus, we believe that the ET-1 stroke model is superior to mimic the key aspects of stroke-induced injury.

Microinjection of ET-1 (400 pmol) is widely used and has been reported as a simple and reproducible method of focal ischemia that in rats produces lesions 14–20 mm^3^ in size.^43, 70^ In contrast, in mice the use of ET-1 has been more limited and the results are more variable.^44, 45, 51^ In this study we used a total amount of 4 μg (1600 pmol) of ET-1, compared with the administration of 1 μg (400 pmol) that has been used in mice by others.^35, 39^ Consistent with previous reports,^51, 71^ our pilot studies confirmed that microinjection of 1 μg of ET-1 produced minimal lesions in C57/BL6 mice (data not shown). Our preliminary studies showed that to optimize consistent drug delivery and avoid denaturation and incomplete solubility that could account for inconsistent results, a high concentration of ET-1 is completely solubilized in sterile water using sonication at 4 °C for 15–20 min, rather than in saline by vigorous vortexing (data not shown). Our results indicate that higher concentrations of ET-1 are necessary to obtain consistent and reproducible focal ischemic lesions in mice compared with rats.

To validate the PSD phenotype in sub-chronic and chronic phases following stroke, we have followed best practice guidelines for testing mouse behavior, which includes using: (i) more than one behavioral test; (ii) using tests that have construct and face validity, as well as outcomes that are objective, reproducible and sensitive enough to detect a long-term deficit.^56, 72^ The ET-1-induced ischemic lesion of the left mPFC produced a significant anxiety phenotype detected in all four tests: EPM, OF, LD and NSF; and a behavioral despair phenotype in both the TST and FST. Thus this is a robust phenotype that can be detected in all measures used to detect anxiety and depressive-like behaviors.

Recently, large bilateral ischemic lesions to the PFC in the rat and mouse have been found to induce learning and memory deficits.^73, 74^ Given the role of the mPFC in cognitive function^64^ and the association of impaired cognition with anxiety and depression in left hemisphere clinical stroke,^12^ it would be interesting to test whether our unilateral lesion of the left mPFC is sufficient to elicit cognitive deficits. Results of MCAO ischemia in rodents have suggested a laterality with increased locomotor activity after right MCAO and anxiety after left MCAO, but others have not seen increased locomotion or anxiety.^26^ This inconsistency may reflect the variability of MCAO strokes in different studies. A recent study in rats found that bilateral ET-1-induced lesion of the rat mPFC showed no change or reduced anxiety in the light–dark or OF test, but did show cognitive impairment.^73^ The robust behavioral phenotype obtained with discrete unilateral lesion in the left mPFC compared with larger bilateral lesions, could suggest that anxiety and depression phenotypes may be more pronounced for unilateral lesions, but this remains to be further tested.

### Cellular changes at PSD lesion site fail to rescue behavior

An additional goal of this study was to establish a stroke model with a persistent behavioral phenotype to study and optimize recovery from PSD. In many animal models, lesion size has been used as a measure of stroke severity without functional assessment.^26^ On the other hand, clinical studies focus on functional recovery outcomes following stroke, which are not often correlated with infarct volume.^75, 76, 77^ Our ET-1-induced stroke model displays a strong depression and anxiety phenotype that persists for at least 6 weeks post stroke, while at this time the lesion site in the left mPFC is barely detectable by CV staining. In addition, by 6 weeks post stroke the microglia that were observed at 48 h post stroke had largely vacated the lesion and a large number of neurons were found in the previous location of the lesion. Shrinkage of the lesion site could explain the repopulation, but tissue shrinkage in response to ischemia appears to involve brain atrophy.^78^ The initial glial reaction suggests that an active remodeling of the lesion may be occurring to allow neurons to migrate from adjacent tissue (penumbra) or from neurogenic sites, although mainly newborn glia reach the stroke site.^79^ This neuronal repopulation is in contrast to what is found in ET-1 ischemic lesions that are 10- to 500-fold larger, in which lesion site forms a glial scar populated by astrocytes and microglia that may prevent repopulation by neurons.^80^ Furthermore, most studies examine lesions at earlier times than 6 weeks post stroke that may not be sufficient to observe refilling of the lesion, as we observed no change in the lesion size at 2 weeks post stroke. Despite the presence of neurons within the lesion site, the stroke mice show persistent anxiety and depression behavior at 6 weeks post stroke.

The observed dissociation between lesion size and behavioral recovery in this PSD preclinical model indicates that the neurons within the lesion site are not correctly integrated in neural networks to restore behavior. This result highlights the importance of measuring functional outcomes of recovery rather than lesion size as an index of recovery in preclinical animal studies, similar to what is done in clinical stroke studies.^77, 81^ Because after 6 weeks the behavioral phenotype did not improve, the NeuN-expressing neurons that infiltrated the stroke lesion did not appear to be integrated into the behavioral circuitry, and this could be verified using electrophysiological analysis. Therapeutic strategies to enhance integration of these neurons and promote recovery could be assessed in future studies. In addition, altered activity through diaschisis of brain regions that are connected to the mPFC may contribute to the behavioral phenotype,^82^ which remains to be addressed in this stroke model. By identifying stroke-induced activity changes in the anxiety-depression circuitry over time, it may be possible to modify abnormal activity to normalize behavior. In this regard the impact chronic antidepressant treatment on the behavioral phenotype and brain activity may help elucidate whether this PSD model displays a similar or different treatment response compared with models of stress-induced depression.

## Conclusion

Taken together, we have shown that injection of ET-1 into the left mPFC of mice produces a robust model of PSD associated with persistent anxiety- and depression-like behavior suitable for preclinical work aimed to improve PSD recovery outcomes.

## Figures and Tables

**Table 1:** Quantification of cellular changes in the ischemic lesion site over time

	*Left cingulate gyrus*		*Left pre/infra-limbic cortex*	
	*48* *h*	*6 weeks*	*48* *h*	*6 weeks*
*IBa-1*	63 ± 4.4	19.7 ± 2.7***	113 ± 10.0	23.3 ± 3.5***
*GFAP*	1.3 ± 0.3	19.0 ± 3.8**	46.3 ± 6.1	7.0 ± 1.5**
*NeuN*	5.0 ± 1.5	266 ± 22**	31.7 ± 4.5	311 ± 40**

Sections of the left medial prefrontal cortex from mice at 48 h (*n*=3) or 6 weeks (*n*=4) post stroke were stained with anti-IBa-1 (microglia), GFAP (astrocyte) and NeuN (neurons), as shown in Figure 3. The number of cells per section within the left CG or pre- and infralmbic cortex were quantified and presented as mean values±s.e.m. **P<0.01; ***P<0.001, compared with 48 h.

## References

[bib1] Esparrago Llorca G, Castilla-Guerra L, Fernandez Moreno MC, Ruiz Doblado S, Jimenez Hernandez MD. Post-stroke depression: an update. Neurologia 2015; 30: 23–31. 22901370 10.1016/j.nrl.2012.06.008

[bib2] Williams LS. Depression and stroke: cause or consequence? Semin Neurol 2005; 25: 396–409. 16341996 10.1055/s-2005-923534

[bib3] Carota A, Berney A, Aybek S, Iaria G, Staub F, Ghika-Schmid F et al. A prospective study of predictors of poststroke depression. Neurology 2005; 64: 428–433. 15699370 10.1212/01.WNL.0000150935.05940.2D

[bib4] Kotila M, Numminen H, Waltimo O, Kaste M. Depression after stroke: results of the FINNSTROKE Study. Stroke 1998; 29: 368–372. 9472876 10.1161/01.str.29.2.368

[bib5] Paolucci S, Gandolfo C, Provinciali L, Torta R, Toso V. The Italian multicenter observational study on post-stroke depression (DESTRO). J Neurol 2006; 253: 556–562. 16767539 10.1007/s00415-006-0058-6

[bib6] Campbell Burton CA, Holmes J, Murray J, Gillespie D, Lightbody CE, Watkins CL et al. Interventions for treating anxiety after stroke. Cochrane Database Syst Rev 2011; 12: Cd008860. 22161439 10.1002/14651858.CD008860.pub2

[bib7] Galligan NG, Hevey D, Coen RF, Harbison JA. Clarifying the associations between anxiety, depression and fatigue following stroke. J Health Psychol 2015; 1–9. 10.1177/1359105315587140 26124087

[bib8] Almeida OP, Xiao J. Mortality associated with incident mental health disorders after stroke. Aust NZ J Psychiatry 2007; 41: 274–281. 10.1080/00048670601172772 17464709

[bib9] Wolfe CD, Crichton SL, Heuschmann PU, McKevitt CJ, Toschke AM, Grieve AP et al. Estimates of outcomes up to ten years after stroke: analysis from the prospective South London Stroke Register. PLoS Med 2011; 8: e1001033. 21610863 10.1371/journal.pmed.1001033 PMC3096613

[bib10] Whyte EM, Mulsant BH, Rovner BW, Reynolds CF. Preventing depression after stroke. Int Rev Psychiatry 2006; 18: 471–481. 17085365 10.1080/09540260600935470

[bib11] Hakim AM. Depression, strokes and dementia: new biological insights into an unfortunate pathway. Cardiovasc Psychiatry Neurol 2011; 2011: 649629. 22216404 10.1155/2011/649629 PMC3246693

[bib12] Barker-Collo SL. Depression and anxiety 3 months post stroke: Prevalence and correlates. Arch Clin Neuropsychol 2007; 22: 519–531. 17462857 10.1016/j.acn.2007.03.002

[bib13] Nys GM, van Zandvoort MJ, van der Worp HB, de Haan EH, de Kort PL, Jansen BP et al. Early cognitive impairment predicts long-term depressive symptoms and quality of life after stroke. J Neurol Sci 2006; 247: 149–156. 16716359 10.1016/j.jns.2006.04.005

[bib14] Thomas SA, Lincoln NB. Factors relating to depression after stroke. Br J Clin Psychol 2006; 45: 49–61. 16480566 10.1348/014466505X34183

[bib15] Mayo NE, Korner-Bitensky NA, Becker R. Recovery time of independent function post-stroke. Am J Phys Med Rehabil 1991; 70: 5–12. 1994971 10.1097/00002060-199102000-00003

[bib16] Goodwin RD, Devanand DP. Stroke, depression, and functional health outcomes among adults in the community. J Geriatr Psychiatry Neurol 2008; 21: 41–46. 18287169 10.1177/0891988707311041

[bib17] Wulsin L, Alwell K, Moomaw CJ, Lindsell CJ, Kleindorfer DO, Woo D et al. Comparison of two depression measures for predicting stroke outcomes. J Psychosom Res 2012; 72: 175–179. 22325695 10.1016/j.jpsychores.2011.11.015 PMC3742310

[bib18] Atteih S, Mellon L, Hall P, Brewer L, Horgan F, Williams D et al. Implications of stroke for caregiver outcomes: findings from the ASPIRE-S study. Int J Stroke 2015; 10: 918–923. 26061711 10.1111/ijs.12535

[bib19] Ghose SS, Williams LS, Swindle RW. Depression and other mental health diagnoses after stroke increase inpatient and outpatient medical utilization three years poststroke. Med Care 2005; 43: 1259–1264. 16299438 10.1097/01.mlr.0000185711.50480.13

[bib20] Husaini B, Levine R, Sharp L, Cain V, Novotny M, Hull P et al. Depression increases stroke hospitalization cost: an analysis of 17,010 stroke patients in 2008 by race and gender. Stroke Res Treat 2013; 2013: 846732. 23555070 10.1155/2013/846732 PMC3608101

[bib21] Wu RH, Feng C, Xu Y, Hua T, Liu XY, Fang M. Late-onset depression in the absence of stroke: associated with silent brain infarctions, microbleeds and lesion locations. Int J Med Sci 2014; 11: 587–592. 24782647 10.7150/ijms.8025 PMC4003543

[bib22] Leary MC, Saver JL. Annual incidence of first silent stroke in the United States: a preliminary estimate. Cerebrovasc Dis 2003; 16: 280–285. 12865617 10.1159/000071128

[bib23] Vermeer SE, Longstreth WT Jr, Koudstaal PJ. Silent brain infarcts: a systematic review. Lancet Neurol 2007; 6: 611–619. 17582361 10.1016/S1474-4422(07)70170-9

[bib24] Mead GE, Hsieh CF, Lee R, Kutlubaev MA, Claxton A, Hankey GJ et al. Selective serotonin reuptake inhibitors (SSRIs) for stroke recovery. Cochrane Database Syst Rev 2012; 11: Cd009286. 23152272 10.1002/14651858.CD009286.pub2 PMC6465036

[bib25] Trivedi MH, Rush AJ, Wisniewski SR, Nierenberg AA, Warden D, Ritz L et al. Evaluation of outcomes with citalopram for depression using measurement-based care in STAR*D: implications for clinical practice. Am J Psychiatry 2006; 163: 28–40. 16390886 10.1176/appi.ajp.163.1.28

[bib26] Kronenberg G, Gertz K, Heinz A, Endres M. Of mice and men: modelling post-stroke depression experimentally. Br J Pharmacol 2014; 171: 4673–4689. 24838087 10.1111/bph.12775 PMC4209937

[bib27] McCann SK, Irvine C, Mead GE, Sena ES, Currie GL, Egan KE et al. Efficacy of antidepressants in animal models of ischemic stroke: a systematic review and meta-analysis. Stroke 2014; 45: 3055–3063. 25184357 10.1161/STROKEAHA.114.006304

[bib28] Robinson RG, Jorge RE. Post-stroke depression: a review. Am J Psychiatry 2016; 173: 221–231. 26684921 10.1176/appi.ajp.2015.15030363

[bib29] Astrom M, Adolfsson R, Asplund K. Major depression in stroke patients. A 3-year longitudinal study. Stroke 1993; 24: 976–982. 8322398 10.1161/01.str.24.7.976

[bib30] Narushima K, Kosier JT, Robinson RG. A reappraisal of poststroke depression, intra- and inter-hemispheric lesion location using meta-analysis. J Neuropsychiatry Clin Neurosci 2003; 15: 422–430. 14627768 10.1176/jnp.15.4.422

[bib31] Wei N, Yong W, Li X, Zhou Y, Deng M, Zhu H et al. Post-stroke depression and lesion location: a systematic review. J Neurol 2015; 262: 81–90. 25308633 10.1007/s00415-014-7534-1

[bib32] de Groot JC, de Leeuw FE, Oudkerk M, Hofman A, Jolles J, Breteler MM. Cerebral white matter lesions and depressive symptoms in elderly adults. Arch Gen Psychiatry 2000; 57: 1071–1076. 11074873 10.1001/archpsyc.57.11.1071

[bib33] Gong Q, He Y. Depression, neuroimaging and connectomics: a selective overview. Biol Psychiatry 2015; 77: 223–235. 25444171 10.1016/j.biopsych.2014.08.009

[bib34] Liao Y, Huang X, Wu Q, Yang C, Kuang W, Du M et al. Is depression a disconnection syndrome? Meta-analysis of diffusion tensor imaging studies in patients with MDD. J Psychiatry Neurosci 2013; 38: 49–56. 22691300 10.1503/jpn.110180 PMC3529219

[bib35] Brookes RL, Herbert V, Lawrence AJ, Morris RG, Markus HS. Depression in small-vessel disease relates to white matter ultrastructural damage, not disability. Neurology 2014; 83: 1417–1423. 25230999 10.1212/WNL.0000000000000882 PMC4206159

[bib36] Flaster M, Sharma A, Rao M. Poststroke depression: a review emphasizing the role of prophylactic treatment and synergy with treatment for motor recovery. Top Stroke Rehabil 2013; 20: 139–150. 23611855 10.1310/tsr2002-139

[bib37] Klein J, Winter C, Coquery N, Heinz A, Morgenstern R, Kupsch A et al. Lesion of the medial prefrontal cortex and the subthalamic nucleus selectively affect depression-like behavior in rats. Behav Brain Res 2010; 213: 73–81. 20434489 10.1016/j.bbr.2010.04.036

[bib38] Covington HE 3rd, Lobo MK, Maze I, Vialou V, Hyman JM, Zaman S et al. Antidepressant effect of optogenetic stimulation of the medial prefrontal cortex. J Neurosci 2010; 30: 16082–16090. 21123555 10.1523/JNEUROSCI.1731-10.2010 PMC3004756

[bib39] Vialou V, Bagot RC, Cahill ME, Ferguson D, Robison AJ, Dietz DM et al. Prefrontal cortical circuit for depression- and anxiety-related behaviors mediated by cholecystokinin: role of DeltaFosB. J Neurosci 2014; 34: 3878–3887. 24623766 10.1523/JNEUROSCI.1787-13.2014 PMC3951691

[bib40] Warner-Schmidt J. Treating the brain deep down: short-circuiting depression. Nat Med 2013; 19: 680–681. 23744149 10.1038/nm.3215

[bib41] Belzung C, Turiault M, Griebel G. Optogenetics to study the circuits of fear- and depression-like behaviors: a critical analysis. Pharmacol Biochem Behav 2014; 122: 144–157. 24727401 10.1016/j.pbb.2014.04.002

[bib42] Fuxe K, Cintra A, Andbjer B, Anggard E, Goldstein M, Agnati LF. Centrally administered endothelin-1 produces lesions in the brain of the male rat. Acta Physiol Scand 1989; 137: 155–156. 2678898 10.1111/j.1748-1716.1989.tb08734.x

[bib43] Windle V, Corbett D. Fluoxetine and recovery of motor function after focal ischemia in rats. Brain Res 2005; 1044: 25–32. 15862786 10.1016/j.brainres.2005.02.060

[bib44] Tennant KA, Jones TA. Sensorimotor behavioral effects of endothelin-1 induced small cortical infarcts in C57BL/6 mice. J Neurosci Methods 2009; 181: 18–26. 19383512 10.1016/j.jneumeth.2009.04.009 PMC6667280

[bib45] Roome RB, Bartlett RF, Jeffers M, Xiong J, Corbett D, Vanderluit JL. A reproducible Endothelin-1 model of forelimb motor cortex stroke in the mouse. J Neurosci Methods 2014; 233: 34–44. 24915635 10.1016/j.jneumeth.2014.05.014

[bib46] Belzung C, Griebel G. Measuring normal and pathological anxiety-like behaviour in mice: a review. Behav Brain Res 2001; 125: 141–149. 11682105 10.1016/s0166-4328(01)00291-1

[bib47] Crawley JN. What is Wrong with My Mouse?: Behavioral Phenotyping of Transgenic and Knockout Mice. Wiley-Liss: Hoboken, NJ, USA, 2007.

[bib48] Franklin K, Paxinos G. The Mouse Brain in Stereotaxic Coordinates with CDROM. Academic Press: New York, NY, USA, 2007.

[bib49] Tureyen K, Vemuganti R, Sailor KA, Dempsey RJ. Infarct volume quantification in mouse focal cerebral ischemia: a comparison of triphenyltetrazolium chloride and cresyl violet staining techniques. J Neurosci Methods 2004; 139: 203–207. 15488233 10.1016/j.jneumeth.2004.04.029

[bib50] Windle V, Szymanska A, Granter-Button S, White C, Buist R, Peeling J et al. An analysis of four different methods of producing focal cerebral ischemia with endothelin-1 in the rat. Exp Neurol 2006; 201: 324–334. 16740259 10.1016/j.expneurol.2006.04.012

[bib51] Horie N, Maag AL, Hamilton SA, Shichinohe H, Bliss TM, Steinberg GK. Mouse model of focal cerebral ischemia using endothelin-1. J Neurosci Methods 2008; 173: 286–290. 18621079 10.1016/j.jneumeth.2008.06.013 PMC2572560

[bib52] Carobrez AP, Bertoglio LJ. Ethological and temporal analyses of anxiety-like behavior: the elevated plus-maze model 20 years on. Neurosci Biobehav Rev 2005; 29: 1193–1205. 16084592 10.1016/j.neubiorev.2005.04.017

[bib53] Hascoet M, Bourin M. A new approach to the light/dark test procedure in mice. Pharmacol Biochem Behav 1998; 60: 645–653. 9678648 10.1016/s0091-3057(98)00031-8

[bib54] Santarelli L, Saxe M, Gross C, Surget A, Battaglia F, Dulawa S et al. Requirement of hippocampal neurogenesis for the behavioral effects of antidepressants. Science 2003; 301: 805–809. 12907793 10.1126/science.1083328

[bib55] Castagne V, Moser P, Roux S, Porsolt RD. Rodent models of depression: forced swim and tail suspension behavioral despair tests in rats and mice. Curr Protoc Neurosci 2011; Chapter 8: Unit 8.10 A. 10.1002/0471142301.ns0810as55 21462162

[bib56] Crawley JN. Behavioral phenotyping strategies for mutant mice. Neuron 2008; 57: 809–818. 18367082 10.1016/j.neuron.2008.03.001

[bib57] Metz GA, Whishaw IQ. The ladder rung walking task: a scoring system and its practical application. J Vis Exp 2009; 28: 1–4. 10.3791/1204 PMC2796662 19525918

[bib58] Cryan JF, Holmes A. The ascent of mouse: advances in modelling human depression and anxiety. Nat Rev Drug Discov 2005; 4: 775–790. 16138108 10.1038/nrd1825

[bib59] Schallert T, Fleming SM, Leasure JL, Tillerson JL, Bland ST. CNS plasticity and assessment of forelimb sensorimotor outcome in unilateral rat models of stroke, cortical ablation, parkinsonism and spinal cord injury. Neuropharmacology 2000; 39: 777–787. 10699444 10.1016/s0028-3908(00)00005-8

[bib60] Price JL, Drevets WC. Neural circuits underlying the pathophysiology of mood disorders. Trends Cogn Sci 2012; 16: 61–71. 22197477 10.1016/j.tics.2011.12.011

[bib61] Singh A, Black SE, Herrmann N, Leibovitch FS, Ebert PL, Lawrence J et al. Functional and neuroanatomic correlations in poststroke depression: the Sunnybrook Stroke Study. Stroke 2000; 31: 637–644. 10700497 10.1161/01.str.31.3.637

[bib62] Terroni L, Amaro E, Iosifescu DV, Tinone G, Sato JR, Leite CC et al. Stroke lesion in cortical neural circuits and post-stroke incidence of major depressive episode: a 4-month prospective study. World J Biol Psychiatry 2011; 12: 539–548. 21486107 10.3109/15622975.2011.562242 PMC3279135

[bib63] Groenewegen HJ, Wright CI, Uylings HB. The anatomical relationships of the prefrontal cortex with limbic structures and the basal ganglia. J Psychopharmacol 1997; 11: 99–106. 9208373 10.1177/026988119701100202

[bib64] Riga D, Matos MR, Glas A, Smit AB, Spijker S, Van den Oever MC. Optogenetic dissection of medial prefrontal cortex circuitry. Front Syst Neurosci 2014; 8: 230. 25538574 10.3389/fnsys.2014.00230 PMC4260491

[bib65] Yang S, Hua P, Shang X, Cui Z, Zhong S, Gong G et al. A significant risk factor for poststroke depression: the depression-related subnetwork. J Psychiatry Neurosci 2015; 40: 259–268. 25871495 10.1503/jpn.140086 PMC4478059

[bib66] Carmichael ST. Rodent models of focal stroke: size, mechanism, and purpose. NeuroRx 2005; 2: 396–409. 16389304 10.1602/neurorx.2.3.396 PMC1144484

[bib67] Schiller D, Weiner I. Lesions to the basolateral amygdala and the orbitofrontal cortex but not to the medial prefrontal cortex produce an abnormally persistent latent inhibition in rats. Neuroscience 2004; 128: 15–25. 15450350 10.1016/j.neuroscience.2004.06.020

[bib68] Lacroix L, Broersen LM, Weiner I, Feldon J. The effects of excitotoxic lesion of the medial prefrontal cortex on latent inhibition, prepulse inhibition, food hoarding, elevated plus maze, active avoidance and locomotor activity in the rat. Neuroscience 1998; 84: 431–442. 9539214 10.1016/s0306-4522(97)00521-6

[bib69] Glenn MJ, Lehmann H, Mumby DG, Woodside B. Differential fos expression following aspiration, electrolytic, or excitotoxic lesions of the perirhinal cortex in rats. Behav Neurosci 2005; 119: 806–813. 15998202 10.1037/0735-7044.119.3.806

[bib70] Cordova CA, Jackson D, Langdon KD, Hewlett KA, Corbett D. Impaired executive function following ischemic stroke in the rat medial prefrontal cortex. Behav Brain Res 2014; 258: 106–111. 24144544 10.1016/j.bbr.2013.10.022

[bib71] Wang Y, Jin K, Greenberg DA. Neurogenesis associated with endothelin-induced cortical infarction in the mouse. Brain Res 2007; 1167: 118–122. 17669376 10.1016/j.brainres.2007.06.065 PMC2098871

[bib72] Fisher M, Feuerstein G, Howells DW, Hurn PD, Kent TA, Savitz SI et al. Update of the stroke therapy academic industry roundtable preclinical recommendations. Stroke 2009; 40: 2244–2250. 19246690 10.1161/STROKEAHA.108.541128 PMC2888275

[bib73] Livingston-Thomas JM, Jeffers MS, Nguemeni C, Shoichet MS, Morshead CM, Corbett D. Assessing cognitive function following medial prefrontal stroke in the rat. Behav Brain Res 2015; 294: 102–110. 26254877 10.1016/j.bbr.2015.07.053

[bib74] Zhou LY, Wright TE, Clarkson AN. Prefrontal cortex stroke induces delayed impairment in spatial memory. Behav Brain Res 2016; 296: 373–378. 26306825 10.1016/j.bbr.2015.08.022

[bib75] Mark VW, Taub E, Perkins C, Gauthier L, Uswatte G. MRI infarction load and CI therapy outcomes for chronic post-stroke hemiparesis. Restor Neurol Neurosci 2008; 26: 13–33. 18431003

[bib76] Riley JD, Le V, Der-Yeghiaian L, See J, Newton JM, Ward NS et al. Anatomy of stroke injury predicts gains from therapy. Stroke 2011; 42: 421–426. 21164128 10.1161/STROKEAHA.110.599340 PMC3026869

[bib77] Page SJ, Gauthier LV, White S. Size doesn't matter: cortical stroke lesion volume is not associated with upper extremity motor impairment and function in mild, chronic hemiparesis. Arch Phys Med Rehabi 2013; 94: 817–821. 10.1016/j.apmr.2013.01.010 PMC3733358 23337427

[bib78] Kraemer M, Schormann T, Hagemann G, Qi B, Witte OW, Seitz RJ. Delayed shrinkage of the brain after ischemic stroke: preliminary observations with voxel-guided morphometry. J Neuroimaging 2004; 14: 265–272. 15228769 10.1177/1051228404264950

[bib79] Li L, Harms KM, Ventura PB, Lagace DC, Eisch AJ, Cunningham LA. Focal cerebral ischemia induces a multilineage cytogenic response from adult subventricular zone that is predominantly gliogenic. Glia 2010; 58: 1610–1619. 20578055 10.1002/glia.21033 PMC2919586

[bib80] Abeysinghe HC, Bokhari L, Dusting GJ, Roulston CL. Brain remodelling following endothelin-1 induced stroke in conscious rats. PLoS One 2014; 9: e97007. 24809543 10.1371/journal.pone.0097007 PMC4029108

[bib81] Chen CL, Tang FT, Chen HC, Chung CY, Wong MK. Brain lesion size and location: effects on motor recovery and functional outcome in stroke patients. Arch Phys Med Rehabi 2000; 81: 447–452. 10.1053/mr.2000.3837 10768534

[bib82] Silasi G, Murphy TH. Stroke and the connectome: how connectivity guides therapeutic intervention. Neuron 2014; 83: 1354–1368. 25233317 10.1016/j.neuron.2014.08.052

